# Effects of Estradiol/Micronized Progesterone vs. Conjugated Equine Estrogens/Medroxyprogesterone Acetate on Breast Cancer Gene Expression in Healthy Postmenopausal Women

**DOI:** 10.3390/ijms24044123

**Published:** 2023-02-18

**Authors:** Parameswaran Grace Luther Lalitkumar, Eva Lundström, Birgitta Byström, Dorina Ujvari, Daniel Murkes, Edneia Tani, Gunnar Söderqvist

**Affiliations:** 1 Division for Obstetrics and Gynecology, Department of Women’s and Children’s Health, Karolinska Institutet, SE 17176 Stockholm, Sweden; 2 Department of Pathology, Cytology Karolinska Institutet, SE 17176 Stockholm, Sweden

**Keywords:** breast cancer gene expression, estradiol/micronized progesterone, conjugated equine estrogens/medroxyprogesterone acetate, healthy postmenopausal women, core needle biopsies, menopausal hormone treatment and breast cancer risk

## Abstract

Recent studies suggest estradiol (E_2_)/natural progesterone (P) confers less breast cancer risk compared with conjugated equine estrogens (CEE)/synthetic progestogens. We investigate if *differences in the regulation of breast cancer-related gene expression* could provide some explanation. This study is a subset of a monocentric, 2-way, open observer-blinded, phase 4 randomized controlled trial on healthy postmenopausal women with climacteric symptoms (ClinicalTrials.gov; EUCTR-2005/001016-51). Study medication was two 28-day cycles of sequential hormone treatment with oral 0.625 mg CEE and 5 mg of oral medroxyprogesterone acetate (MPA) or 1.5 mg E_2_ as percutaneous gel/day with the addition of 200 mg oral micronized P. MPA and P were added days 15–28/cycle. Material from two core-needle breast biopsies in 15 women in each group was subject to quantitative PCR (Q-PCR). The primary endpoint was a change in breast carcinoma development gene expression. In the first eight consecutive women, RNA was extracted at baseline and after two months of treatment and subjected to microarray for 28856 genes and Ingenuity Pathways Analysis (IPA) to identify risk factor genes. Microarray analysis showed 3272 genes regulated with a fold-change of >±1.4. IPA showed 225 genes belonging to mammary-tumor development function: 198 for CEE/MPA vs. 34 for E_2_/P. Sixteen genes involved in mammary tumor inclination were subject to Q-PCR, inclining the CEE/MPA group towards an increased risk for breast carcinoma compared to the E_2_/P group at a very high significance level (*p* = 3.1 × 10^−8^, *z*-score 1.94). The combination of E_2_/P affected breast cancer-related genes much less than CEE/MPA.

## 1. Introduction

Menopausal hormone treatment (MHT) is used for the alleviation of climacteric symptoms but has been associated with an increased risk of breast cancer after long-term treatment [1,2,3,4,5,6]. Since breast cancer is so common, even a small increase in the odds ratio (OR) will have a great impact on the absolute number of breast cancer cases. The increased risk of developing breast cancer in women using CEE in combination with the synthetic progestogen MPA was one of the reasons for the premature termination of the CEE-MPA arm in the WHI study [1]. Since then, many women have discontinued their MHT despite sometimes severe climacteric symptoms.

Sex hormones in different combinations, doses, regimens, and durations may have various effects on the breast. Our group and others have shown that CEE in combination with the synthetic progestogen MPA induces a different genetic and proliferative response in breast cells in vivo and in vitro than when given together with natural micronized progesterone [6,7,8]. In a previous study by our group at the Karolinska Institutet, mitogenic activity was found to increase when oral estradiol was combined with noretisterone acetate as well as with dienogest [9]. The use of natural and topical MHT seems to have less impact on breast cancer risk. In the French E3N cohort, women on estrogen in combination with different synthetic progestogens conferred an increased risk of breast cancer compared to women taking estrogen and natural progesterone formulations [10,11,12]. We used a 2.0 mm core needle biopsy (CNB) to increase the cell amount [13]. All samples were retrieved before and at the end of two months of treatment, making every subject under their own control. The stimulation of proliferation in breast cells can already be seen after two months of treatment [8,14,15,16].

The purpose of this study was to evaluate any differences in the change in expression of several genes relevant to mammary tumor development in the treatment groups.

## 2. Results

Fifteen women receiving CEE/MPA and fifteen women receiving(E_2_)/P were the subsets for gene expression analysis. Patient demographics at screening are illustrated in Table 1.

### 2.1. Serum Hormones

Serum hormone levels at baseline and after two months of treatment were assessed on the same day as the CNBs. Estradiol (E_2_) (*p* < 0.01) and sex hormone binding globulin (SHBG) (*p* < 0.05) increased significantly during treatment in both treatment groups. Free testosterone (fT) decreased significantly only for women on CEE/MPA (*p* = 0.002). Insulin growth factor 1 (IGF-1) (*p* < 0.01) and insulin growth factor binding protein 3 (IGFBP-3) (*p* = 0.01) decreased significantly in both groups.

### 2.2. Microarray

For the eight patients (CEE/MPA *n* = 4 and (E_2_)/P *n* = 4), with biopsies before and on one of the days 54–56 of treatment, the expression values of 28,856 genes were further analyzed.

During treatment, 3272 [2735 unique for CEE/MPA; 340 for (E_2_)/P; and 197 common] genes were changed with a fold change of <−1.4 or >1.4 and subject to further analysis with IPA.

Among the 3272 genes, IPA classified 225 genes as “affecting mammary tumor development”, 198 genes for CEE/MPA, and 34 for (E_2_)/P. For 18/225 genes, it was indicated in the IPA database whether up- or down-regulation would increase mammary tumor inclination: Fourteen of these eighteen genes were concluded to increase mammary tumor development more for CEE/MPA than for (E_2_)/P. The corresponding figure for tumor inclination was 4/18 genes more for (E_2_)/P than CEE/MPA. For 11 of the 18 genes, microarray data indicated enough mRNA content in the normal breast tissue to be further assessed with Q-PCR. In addition, we chose to study another five genes of interest according to the literature: the MKi-67, Bcl-2, PGR (B), IGF-1, and cERB-B3 genes (Table 2).

### 2.3. Q-PCR

The change in Q-PCR expression of the 16 genes (11 + 5), with sufficient mRNA in both specimens, for the 30 patients (15 in each treatment group), is given in Table 3.

**Figure 1:**
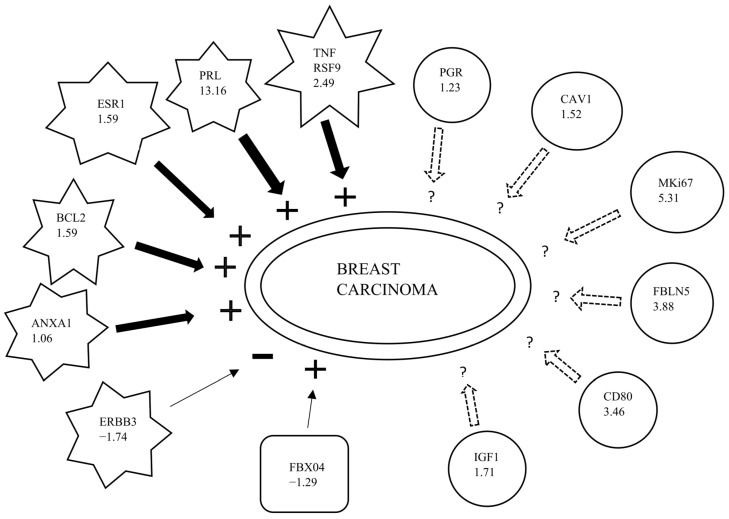
Fold change ratios (no’: s within symbols) for CEE/MPA vs. (E_2_)/P for thirteen genes affecting breast carcinoma. Star-shaped symbols represent genes increasing breast carcinoma when activated. Plus: **+—**sign represents net effect of increasing breast carcinoma inclination. Minus: **−**sign represents net effect decreasing breast carcinoma inclination. Rectangle represents gene with protective effect against breast carcinoma. Thick black arrows represent positive FC ratios. Thin black arrows represent negative FC ratios. Dotted arrows indicate findings inconsistent with state of downstream molecule. The biological function “breast carcinoma” was augmented more for CEE/MPA than for (E_2_)/P at a very high significance level (*p* = 3.1 × 10^−8^, *z*-score = 1.94). (Adapted information from IPA database, Ingenuity Systems).

Between treatments, FC ratios from Q-PCR of the 16 genes as specified in Table 1 were re-uploaded to IPA and compared. The biological function “breast carcinoma” was augmented more for CEE/MPA than for (E_2_)/P at a very high significance level (*p* = 3.08 × 10^−8^, *z*-score = 1.94). Moreover, 13 out of the 16 genes were involved in this biological function, where 6/13 genes were shown to augment the IPA- function “breast carcinoma” more for CEE/MPA than (E_2_)/P vs. 1/13 genes to augment this function more for (E_2_)/P than CEE/MPA (Figure 1 and Table 4).

A significant increase in MKi-67 and IGF-1 gene expression (*p* < 0.05) was found in the CEE/MPA group only. In the (E_2_)/P group, the prolactin and Bcl-2 genes were down-regulated (*p* < 0.05), (Table 3).

For the eight subjects who were analyzed by both microarray and Q-PCR, we found a high correlation between the methods for the mRNA expression fold changes of the sixteen genes given in Table 1 and Table 3 (Rs = 0.5; *p* = 0.005).

## 3. Discussion

This is the first prospective randomized study to describe the effects on genetic expression in mammary tissue from healthy postmenopausal women during treatment with sequential MHT with either natural (E_2_)/P or synthetic CEE/MPA. The effects of (E_2_), (E_2_)/NETA, and tibolone have previously been evaluated [17]. Repeated CNBs allowed the women to act as their own controls and made it possible to measure the actual change incurred by treatment for every investigated gene. The use of a housekeeping gene in each sample and the same primers for all genes assessed by microarray and PCR improved assessment accuracy, as evidenced by the high correlation between methods. Microarray data showed five times more genes to be affected by CEE/MPA as compared to (E_2_)/P treatment.

IPA Upstream Regulator Analysis (URA) and Downstream Effects Analysis (DEA) are powerful tools to assess the activity of a transcriptional regulator as well as biological functions and diseases that are downstream of genes with altered expression during treatment. The information in IPA is collected from numerous experimental systems into a continuously updated knowledge base [18].

We found a remarkable difference between the two alternatives for sequential MHT in healthy postmenopausal women. Between-treatment FCs from Q-PCR for the 16 genes as specified in Table 1 were re-uploaded to IPA and compared. The biological function “breast carcinoma” was augmented more for CEE/MPA than for (E_2_)/P at a very high significance level (*p* = 3.08 × 10^−8^), indicating a striking difference between the two MHTs for this important adverse effect.

The CEE/MPA preparation conferred an augmented breast cancer risk in the WHI study. This study may contribute to some explanation for this risk, based on our results concerning breast cancer gene expression. The French E3N-cohort data indicating MHT with(E_2_)/P as less detrimental is also in concordance with our current findings [6,7,8,19,20,21,22]. Although these data seem very favorable for (E_2_)//P, in this study there was also a marked variation in response between individual women [8,9,15]. A few women, tentatively more sensitive to hormones than the majority, had a proliferative response also during E_2_/P treatment and a concomitant MKi-67 gene activation.

An anti-proliferative drug in the normal breast found previously was the anti-progesterone mifepristone, where a significant down-regulation of Ki-67 protein in the breast was seen in premenopausal women treated for leiomyoma. In that study, material from an FNA biopsy was not sufficient for gene expression studies [23].

The present gene data from the current subset of 15 + 15 patients were compared to previously reported findings on proliferation and apoptosis using the Ki-67 and Bcl-2 antibodies in the same clinical material [8]. In the total subset material, there was a highly significant correlation (Rs = 0.67; *p* = 0.026) between the difference in expression of the MKi-67 gene and the increase in the percentage of Ki-67- MIB1-positive cells during treatment. A positive correlation between the change in IGF-1 gene expression and the Ki-67 MIB1 protein was found in the CEE/MPA group but not in the E_2_/P group. This correlation, apparent only for CEE/MPA, is interesting. It indicates the IGF-1 gene as one of the candidate genes for possible mechanisms behind the observed proliferative effects induced by this treatment. Previously, a significant correlation between IGF-1 mRNA and Ki-67 protein was seen in women during hormonal contraception with ethinylestradiol/levonorgestrel. High IGF-1 levels were found to be a risk factor for breast cancer in epidemiologic studies as well as a mitogen for many breast cancer cell lines [24,25,26].

We also found a marked down-regulation of the proliferative prolactin gene in the E_2_/P group, which was not apparent during CEE/MPA treatment. The drop in endogenous estradiol and progesterone at parturition induces prolactin gene activation, stimulating lactation [27]. Obviously, in postmenopausal women with low endogenous E_2_ and P levels, natural E_2_/P treatment induces the logical opposite effect of down-regulating the prolactin gene. Here we find that CEE/MPA treatment is devoid of this physiologic capacity [28,29].

The anti-apoptotic Bcl-2 gene was down-regulated by E_2_/P, which was not evident for CEE/MPA, where the MKi-67 gene increased during treatment [30].

The significant positive correlation between the MKi-67 gene and Ki-67 protein expression for the total material supports the opinion that proliferative responses on both MHTs coincide with increased Ki-67 protein production and not with reduced protein degradation [31].

## 4. Materials and Methods

### 4.1. Study Design and Patients

We performed a monocentric, 2-way, open (observer-blinded), phase 4 randomzsed controlled trial (investigator-sponsored study) at the Clinical Research Unit of the Department of Obstetrics and Gynecology at the Karolinska University Hospital/Institutet, and in the Unilabs Mammography Department, Capio St Göran’s Hospital, in Stockholm, Sweden.

Postmenopausal, apparently healthy women, non-smokers, aged 44 to 66 years without known breast pathology, with normal mammograms and a body mass index (BMI) of 18–30 kg/m^2^ were recruited for the study. They were post-menopausal for at least 12 months before entering the study, as confirmed at the screening by follicle-stimulating hormone (FSH) levels >25 IU/L and E_2_ levels < 90 pmol/L according to the reference values at the Karolinska Hospital accredited laboratory. The washout period for previous MHT users was three months. The study was approved by the independent ethics committee and the Swedish Medical Products Agency: IRB-2005/762-31, IRB-2013/963-32, and EUCTR-2005/001016-51, respectively. All women gave their written informed consent before inclusion.

### 4.2. Analytical Methods

Circulating sex steroid levels and hormone-binding globulins were quantified by routine hospital methods. Serum concentrations of E_2_ and Sex Hormone Binding Globulin (SHBG) were determined by direct chemiluminescence enzyme immunoassay and total testosterone (T) by direct RIA with a commercial kit (Coat-a-Count Testosterone) (Siemens Healthcare Corporation, Deerfield, IL, USA). Concentrations of free testosterone (fT) were calculated from values for T, SHBG, and a fixed albumin concentration of 40 g/L. IGF-1 was determined by chemiluminescence enzyme immunoassay using a commercial kit (Advantages) obtained from Nichols Products Corporation, San Juan Capistrano, CA, USA. IGF-BP3 was analyzed by ELIZA using a commercial kit obtained from Diagnostic Systems Laboratories Inc., (Webster, TX, USA). The detection limits and within- and between-assay coefficients of variation were for T: 0.1 nmol/L, 6%, and 12%; SHBG: 0.2 nmol/L, 6.5%, and 8.7%; E_2_ (Spectria): 5 pmol/L, 7.4%, and 10.3% (Orion Diagnostica Oy, Espoo, Finland); E_2_ (Immulite): 55 pmol/L, 9.3%, and 10.6% (Diagnostic Products Corporation, Los Angeles, CA, USA), and for P: 0.6 nmol/L, 8.2%, and 9.3%; PRL: 0.04 µg/L, 1.9%, and 3.2%; IGF1: 6 µg/L, 4.8%, and 6.7%; and IGF-BP3: 0.04 µg/L, 9%, and 10%.

### 4.3. Randomisation and Masking

Eligible patients were randomized (1:1) into two groups: MHT with oral CEE/MPA or percutaneous E_2_/oral P. Randomization was done from a list created by a random number generator external to the Department of Obstetrics and Gynecology, and the randomization sequence was kept concealed. The investigators but not the patients were masked to group assignment. The evaluation of IHC, microarray, and PCR data was conducted blinded to treatment.

### 4.4. Study Medication

Seventy-seven healthy women were randomized to sequential hormone therapy with two 28-day cycles of either oral 0.625 mg conjugated equine estrogens (CEE) or 2.5 g 0.06% (1.5 mg E_2_) percutaneous E_2_-gel daily, with the addition of 5 mg of oral medroxyprogesterone acetate (MPA) or 200 mg of oral micronized P, daily, 14/28 days per cycle.

### 4.5. Biopsies

Three CNB specimens from each patient were prepared at baseline and on days 54–56 of treatment. We directed the biopsies stereo-tactically towards areas of the highest mammographic density of the upper outer quadrant of the left breast under local anesthesia on a prone table (LORAD) using a 14G needle and normal breast tissue was procured [32]. Detailed IHC data for Ki-67 and Bcl-2 have been published from the clinical trial [8].

One specimen stored in RNA-Later^®^ was used for this current study, namely, gene expression analyses with microarray and Q-PCR according to the manufacturer’s instructions (Life Technologies Ltd., Paisley, UK).

### 4.6. RNA Extraction, cDNA Synthesis and Microarray

In eight consecutive patients from the clinical trial, four from the CEE/MPA group and four from the E_2_/P group, RNA was extracted in the samples before and at the end of treatment, subject to reverse transcription and amplification, and a cDNA microarray was performed for expression using 28,856 genes derived after background noise reduction on a platform at the Karolinska Bioinformatics and Expression Analysis (BEA) center. Samples were homogenized using a Retsch^®^ tissue mill (Retsch KG, Hahn, Germany) and maintained in liquid nitrogen for 2 min using a shaking frequency of 30/s. Total RNA was first extracted in Trizol^®^ reagent (Life Technologies; Invitrogen Corp. & Applied Biosystems, Inc., Carlsbad, CA, USA) and then stored in a −80 °C freezer until reverse transcription into cDNA was made. Total RNA was purified with the RNeasy Mini Kit (Qiagen GmbH, Hilden, Germany), including treatment with DNase, all steps in accordance with the manufacturer’s instructions.

Total RNA from each sample was used in the standard protocol from NuGen (San Carlos, CA, USA) to label targets. The RNA was reverse transcribed by Affymetrix (Santa Clara, CA, USA) in vitro into single-stranded sense target cDNA, and 1.6 µg per sample was hybridized to Gene Chip^®^ Human Gene 1.0 ST Array Gene Chips according to their expression analysis manual. All samples were of high quality, with an OD 260/280 ratio > 1.8. Genes with a fold change of ≤−1.4 or ≥1.4 were analyzed using Ingenuity Pathways Analysis (IPA) software (Ingenuity© Systems, Inc., www.ingenuity.com May 2018).

### 4.7. Quantitative PCR (Q-PCR)

This study is a subset of the clinical trial. Fifteen consecutive women in each group receiving CEE/MPA and E_2_/P, respectively, were subjected to gene expression analysis with Q-PCR. After reverse-transcription of equal amounts of total RNA, cDNA was formed after pre-treatment with DNAse using Superscript II Reverse Transcriptase (Invitrogen, CA, USA) and Ribonuclease Inhibitor (Promega Corp., Masden, WI, USA).

Gene expression levels were quantified by Q-PCR using Taqman Gene Expression Assays and Taqman Gene Expression Master Mix (Life Technologies) in multiplex reactions. mRNA levels were normalized to the level of endogenous control 18S. Sixteen probes for target genes, eleven genes identified as increasing mammary tumors by the IPA database, and an additional five genes with specific relevance for hormonal effects/risk of cancer in mammary tissue were assessed (Table 1).

We ran the reactions in a Step One 7300 Real-Time multiplex PCR system (Life Technologies). All the reactions contained 10 ng of cDNA in a 25 µL reaction volume. The PCR efficiency with all amplicons was 90–100%, and we performed all determinations in triplicates and included duplicate negative (no-template) and positive controls (Human Placenta Total RNA, Lot No. 030302520J, Ambion Life Technologies, Austin, TX, USA).

Q-PCR reactions were performed on 30 consecutive patients (15 from each treatment group) both at baseline and at the end of the treatment in 96-well optical PCR plates. Target gene TaqMan probes were FAM™ dye-labeled, and 18S cDNA probes were VIC™ dye-labeled; all products, including oligonucleotide primers, were purchased from Applied Biosystems. All plates included 18S rRNA amplification of each sample as an endogenous control for data normalization. The cycling conditions were: 50 °C for 2 min, followed by 95 °C for 10 min, then 40 cycles of 95 °C for 15 s and 60 °C for 1 min. For all sixteen genes, we used the same PCR primers as for the previous microarray.

We analyzed the Q-PCR data using the comparative Ct method, where Ct is the cycle number when the fluorescence first exceeds the threshold, and calculated ΔCt by subtracting the Ct value of the endogenous control from the Ct value of the target gene. This quantification gave us the RQ value [33].

### 4.8. Statistical Analysis

All IPA data were analyzed by Fischer’s exact test within the IPA core analysis program. Power calculations were performed according to earlier studies on Ki-67 protein from FNA biopsies since mRNA studies on normal breast tissue were never previously performed when the study was designed.

Non-IPA data are presented as the arithmetic mean, median, and 25th–75th percentile. Comparisons within the same group of women were carried out by Wilcoxon’s signed-rank test for paired observations and between the two groups by Mann–Whitney U-test. Correlations were performed by Spearman’s rank correlation test. Non-parametric methods were chosen due to the skewed distribution of data. The significance level was set at *p* < 0.05.

## 5. Conclusions

In summary, hormone therapy with CEE/MPA induced a more adverse regulation of genes involved in breast carcinoma inclination compared with E_2_/P in healthy postmenopausal women with moderate climacteric symptoms.

### Limitations

This study was carried out some time ago using microarray, and the results were validated by real-time PCR. However, using the latest technology such as RNA-seq may help us understand the differential gene expression in greater depth.

## Figures and Tables

**Table 1:** Patient demographics.

		CEE/MPA *n* = 15	E_2_/P *n* = 15
Age	Mean	55.8	58.0
	Median	56.0	58.0
	IQR	54.0–60.0	56.0–60.0
BMI	Mean	25.9	24.9
	Median	26.0	24.6
	IQR	24.0–28.0	23.3–26.9
Parity	Mean	1.9	2.1
	Median	2.0	2.0
	IQR	1.0–3.0	1.0–2.8
YSMP	Mean	6.7	7.0
	Median	6.0	5.0
	IQR	3.5–10.0	3.2–10.0

There were no significant differences in any of these parameters at baseline.

**Table 2:** The 16 target genes for PCR.

Affymetrix ID	Gene Abbreviation	Full Gene Name	Taqman Assay ID
8084710	ADIPOQ	Adiponectin, C1Q and collagen domain containing	Hs 00605917_m1
8155849	ANXA1	Annexin A1	Hs 00167549_m1
8056909	ATF2	Activating transcription factor 2	Hs 01095345_m1
8023646	BCl-2	Apoptosis regulator Bcl 2, B-cell lymphoma 2	Hs 00608023_m1
8135594	CAV1	Caveolin 1, coding caveolae protein 22 kDa	Hs 00971716_m1
8089771	CD80	Cluster of Differentiation 80	Hs 00175478_m1
7956120	ERBB3	Receptor tyrosine-kinase erbB-3, HER3 (human epidermal growth factor)	Hs 00176538_m1
8122843	ESR1	Estrogen receptor 1	Hs 00174860_m1
7980908	FBLN5	Fibulin 5	Hs 00197064_m1
8105111	FBXO4	F box protein 4	Hs 00254777_m1
7902227	GADD45A	Growth arrest and DNA damage inducible α	Hs 00169255_m1
7965873	IGF1	Insulin-like growth factor 1	Hs 01547656_m1
7937020	MKI67	Monoclonal antibody Ki 67	Hs 01032443_m1
7951165	PGR	Progesterone receptor	Hs 01556702_m1
8124185	PRL	Prolactin	Hs 00168730_m1
7912145	TNFRSF9	Tumor necrosis factor receptor superfamily, member 9	Hs 00155512_m1

**Table 3:** Effects of the 2 MHTs on fold changes of the 16 genes assessed by Q-PCR. * *p* < 0.05 within group.

Genes	Fold Change CEE/MPA	Fold Change E_2_/P
MKi-	14.16 *	2.67
IGF-1	1.83 *	1.07
PRL	−1.13	−14.88 *
BCl-2	−1.03	−1.64 *
ESR 1	−1.80	−2.86
PGR B	2.47	2.02
TNFSR9	1.03	−2.43
ANXA-1	−1.52	−1.22
CD 80	2.22	−1.56
ATF 2	1.01	−1.11
ADIPOQ	−1.01	−0.52
GADD 45A	1.03	−0.89
FBX 04	−0.18	1.09
CAV 1	−1.16	−1.77
Fibulin 5	3.02	−1.29
cERB B3	−1.70	1.02

**Table 4:** Between treatment effects [a] (CEE/MPA vs. E_2_/P FC ratios) from Q-PCR of the thirteen genes affecting the IPA function “breast carcinoma”. [b]: Literature findings concerning increased expression of the respective gene on “breast carcinoma”.

Affymetrix ID	Genes	Predicted Effect [a]	Fold Change Ratio	Findings [b]
7937020	PRL	Increased	13.60	Increases
7980908	MKi-67	Affected	5.307	Affects
8089771	FBLN5	Affected	3.884	Affects
7912145	CD80	Affected	3.456	Affects
7965873	TNFRSF9	Increased	2.491	Increases
8122843	IGF1	Affected	1.708	Affects
8023646	ESR1	Increased	1.591	Increases
8135594	BCL-2	Increased	1.587	Increases
7951165	CAV1	Affected	1.518	Affects
8155849	PGR	Affected	1.226	Affects
8105111	ANXA1	Increased	1.062	Increases
7956120	FBXO4	Increased	−1.290	Decreases
7956120	ERBB3	Decreased	−1.741	Increases

Adapted from IPA database, Ingenuity Systems.

## Data Availability

Confidential disclosure agreement for the clinical trial from where this subset study is derived prevents public access.
